# Genetic Mapping of the *Gmpgl3* Mutant Reveals the Function of GmTic110a in Soybean Chloroplast Development

**DOI:** 10.3389/fpls.2022.892077

**Published:** 2022-05-26

**Authors:** Hui Yu, Qiushi Wang, Zhirui Zhang, Tao Wu, Xinjing Yang, Xiaobin Zhu, Yongheng Ye, Jiantian Leng, Suxin Yang, Xianzhong Feng

**Affiliations:** ^1^Key Laboratory of Soybean Molecular Design Breeding, Northeast Institute of Geography and Agroecology, Chinese Academy of Sciences, Changchun, China; ^2^College of Advanced Agricultural Sciences, University of Chinese Academy of Sciences, Beijing, China; ^3^Zhejiang Lab, Hangzhou, China

**Keywords:** soybean, *Gmpgl3* mutant, gene mapping, GmTic110a, GmTic20, GmTic40a/b

## Abstract

The generation of oxygen and organic matter in plants mainly depends on photosynthesis, which directly affects plant growth and development. The chloroplast is the main organelle in which photosynthesis occurs. In this study, a *Glycine max* pale green leaf 3-1 (*Gmpgl3-1*) mutant was isolated from the soybean mutagenized population. The *Gmpgl3-1* mutant presented with decreased chlorophyll contents, reduced chloroplast stroma thylakoids, reduced yields, and decreased numbers of pods per plant. Bulked segregant analysis (BSA) together with map-based cloning revealed a single-nucleotide non-synonymous mutation at the 341st nucleotide of the first exon of the chloroplast development-related *GmTic110a* gene. The phenotype of the knockout plants was the same as that of the mutant. The *GmTic110a* gene was highly expressed in the leaves at various developmental stages, and its protein was localized to the inner chloroplast membrane. Split luciferase complementation assays and coimmunoprecipitation (co-IP) experiments revealed that GmTic110a interacted with GmTic20, GmTic40a, and GmTic40b in tobacco leaves. These results indicated that the *GmTic110a* gene plays an important role in chloroplast development.

## Introduction

Plant leaves are the most important tissues for photosynthesis. Chlorophyll is an important pigment involved in photosynthesis in chloroplasts (Waters and Langdale, 2009), and the development of plant chloroplasts positively correlates with the chlorophyll content in leaves (Davis and Fajer, 1979; Wang et al., 2003) and leaf photosynthesis rates (Peng et al., 2008). Mutations in chlorophyll synthesis-related genes can directly or indirectly affect chlorophyll biosynthesis or degradation pathways, leading to the loss of plant chlorophyll, thereby affecting the photosynthesis of plants and causing yellow leaves, albinism, striped leaf spots, purple–brown patches, or other characteristics of chlorophyll-deficient mutants (Awan et al., 1980). To date, researchers have studied chlorophyll-deficient mutants of *Arabidopsis thaliana* (Carol et al., 1999), tobacco (Okabe and Straub, 1977), corn (Lonosky et al., 2004), rice (Ki-Hong et al., 2003), soybean (Stockinger and Walling, 1994), pea (Highkin et al., 1969), wheat (Cao et al., 2006), barley (Preiss and Thornber, 1995) and other plant species. Chlorophyll-deficient mutants usually present with a decreased photosynthesis rate and reduced yields, and death can occur in severe cases.

Plant chloroplasts synthesize important amino acids through photosynthesis and are the main sources of energy for plant cells. Chloroplasts play an important role in plant growth and cellular metabolism (Tiller and Bock, 2014). The transport of substances in and out of chloroplasts depends on the translocon at the outer envelope membrane of chloroplasts (TOC) and the translocon at the inner envelope membrane of chloroplasts (TIC; Soll, 2004; Jocelyn and Paul, 2005; Jarvis, 2008). The TOC and TIC form a complex to facilitate this process. The chloroplast transport proteins on the outer chloroplast membrane identified to date include Toc159 (Waegemann and Soil, 1991; Schnell et al., 1994), Toc34 (Kessler et al., 1994; Schnell et al., 1994), Toc75 (Tranel et al., 1995; Sveshnikova et al., 2000) and Toc64 (Sohrt and Soll, 2000; Becker et al., 2004). Recent extensive studies have significantly updated our understanding of the components and mechanisms of the chloroplast translocon machinery (Kikuchi et al., 2013, 2018; Nakai, 2015, 2018, 2020). These studies have significantly revised the long-accepted “classical” model for chloroplast protein import: In the classical model, Tic110 (Ishida and Terakura, 1987; Inaba et al., 2005), Tic40 (Stahl et al., 1999; Chou et al., 2003), Tic20 (Kouranov and Schnell, 1997; Kasmati et al., 2011), and Tic21 (Vitale et al., 2015) are the main components, but they are not found in the translocon proposed by Nakai (2015, 2018, 2020). In the revised model, the 1-megadalton TIC complex consists of Tic214 (ycf1; de Vries et al., 2007; Bölter and Soll, 2017), Tic100 (Oshima et al., 1987; Ramundo et al., 2020), Tic56 (Köhler et al., 2015, 2016), Tic20 (Kikuchi et al., 2009), and Tic21 (Kouranov and Schnell, 1997; Teng et al., 2006), which functionally and physically cooperate with the ATP-driven import motor YCF2/FTSHI complex (Kikuchi et al., 2013, 2018; Thomson et al., 2020).

Tic110 is an important chloroplast inner membrane protein (Schnell et al., 1994; Inaba et al., 2005; Balsera et al., 2009). It has been reported that the Tic110 protein interacts with several molecular chaperones, such as Hsp93 and Hsp70, to form an inner membrane transport channel scaffold that ensures the successful import of various proteins into the chloroplast to perform cell biological functions (Inaba et al., 2003). Tic110 proteins interact with Tic32 proteins to perform redox functions and to regulate Ca^2+^ homeostasis in the chloroplast (Hormann et al., 2004). In addition, Tsai et al. (2013) reported that Tic110 is most likely a scaffolding component important for protein–protein interactions to recruit other translocon components and chaperones in the stroma (Tsai et al., 2013). In Arabidopsis and soybean, a lack of Tic110 blocks the transport of the inner and outer chloroplast membranes, affecting the development of chloroplasts and resulting in yellow leaves (Inaba et al., 2005; Sandhu et al., 2016). Tic110 assists in the formation of a scaffold for the assembly of the ATP-dependent import motor in the stroma (Richardson and Schnell, 2020). Soybean is an important source of grain and oil. This species is also the main source of high-quality protein for human diets and animal feed. As such, soybean occupies an important position in grain production worldwide. Obstruction of plant chloroplast development could lead to yellow leaves, which severely affects photosynthesis and plant yield; in severe cases, this results in dwarf-type plants or even plants that fail to produce harvestable yields. Therefore, it is of great scientific importance to study the regulatory molecular mechanisms of chloroplast membrane transport proteins.

In this study, we report the characterization of a *Glycine max* pale green leaf mutant (*Gmpgl3-1*). The chloroplast development-related gene *GmTic110a* encodes a chloroplast inner membrane protein. *Gmpgl3-1*, *Gmpgl3-2,* and *Gmpgl3-3* are allelic mutants of the *GmTic110a* gene. The biological function of the *GmTic110a* gene was preliminarily analyzed in this research. We used split luciferase complementation and coimmunoprecipitation (co-IP) analyses, and the results indicated that GmTic110a proteins can interact with other GmTic proteins in tobacco. Our research lays a theoretical foundation for studies of the molecular mechanism underlying soybean chloroplast development.

## Materials and Methods

### Plant Material

All plants used in this study were grown at the Changchun Agricultural Station, Northeast Institute of Geography and Agroecology, Chinese Academy of Science (CAS), China. A *Gmpgl3-1* mutant was isolated from an M_2_ population induced by ethylmethanesulfonate (EMS). The *Gmpgl3-1* mutant was backcrossed to Williams 82 five times from 2014 to 2018 to purify the genetic background of the *Gmpgl3-1* mutant. For protoplast isolation, Arabidopsis seeds of the Columbia ecotype (Col-0) were surface sterilized, vernalized, and then sown on 1/2-strength Murashige and Skoog (MS) media until the seedlings reached the four-leaf stage. Then, the seedlings were grown in pots containing peat moss and vermiculite (1/1, v/v) in a growth chamber under 150 μmol m^−2^ s^−1^ irradiance and a 14 h dark/10 h light photoperiod at 25°C, and the relative humidity was maintained at 60–75%. Leaves were collected from 3- to 4-week-old seedlings for transfection assays.

### Mapping of *GmTic110a via* Bulked Segregant Analysis

Three F_2_ populations derived from a cross between the *Gmpgl3-1* mutant and the Chinese soybean cultivar Hedou 12 were used to map the *GmTic110a* gene. DNA from 50 F_2_ individuals with the *Gmpgl3-1* mutant phenotype and 50 F_2_ individuals with the wild-type phenotype were bulked into mutant and wild-type pools, respectively. Insertion–deletion (INDEL) markers for preliminary mapping were used according to a previously described method (Song et al., 2015). New molecular markers for fine mapping were generated; these markers are listed in Supplementary Table S4. The candidate genomic regions were identified *via* BSA of the F_2_ population at a depth of approximately 30× using an Illumina HiSeq 2000 device (Illumina Inc., San Diego, CA, United States). The Genome Analysis Toolkit (GATK, version 3.8) was used to detect single-nucleotide polymorphisms (SNPs; Mckenna et al., 2010). Genomic regions in which Δ(SNP index) was >0.5 were selected as candidate regions.

### Database Searching and Phylogenetic Analysis

GmTic110a homologs were identified by querying the GmTic110a sequence in the NCBI^1^ database *via* the BLASTP program. Multiple sequence alignments were performed using ClustalX version 2.0 (Larkin et al., 2007) and were manually corrected. The obtained sequence was used as input to construct an unrooted phylogenetic tree with the neighbor-joining algorithm *via* the Molecular Evolutionary Genetics Analysis version 7.0 (MEGA 7.0) phylogenetic program (Sudhir et al., 2016). Bootstrap analysis was performed using 1,000 replicates. The protein motifs of GmTic110a-like genes were subsequently profiled by Multiple Expectation maximization for Motif Elicitation (MEME; Bailey et al., 2009).

### Determination of Pigment Contents and Chlorophyll Fluorescence Analysis

To determine pigment contents, leaves of 21-day-old *Gmpgl3-1* mutants and Williams 82 plants were collected and measured according to a previously reported procedure (Gregor and Marsálek, 2004). The pigment contents were calculated according to the following formulas: chlorophyll a = 13.95^*^A665-6.88^*^A649; chlorophyll b = 24.96^*^A649-7.32^*^A665; and carotenoids = (1,000^*^A470-2.05^*^Ca-114.8^*^Cb)/245. The photosynthesis rate (Pn), stomatal conductance (Gs), intercellular CO_2_ concentration (Mckenna et al.), and transpiration rate (Tr) of the leaves were measured using an LI-6400 photosynthesis system (LI-COR, Lincoln, NE, United States; Yamori et al., 2011), and the initial fluorescence (F0), maximal fluorescence (Fm), and variable fluorescence (Fv) values were measured using an OS-30p chlorophyll fluorometer (Opti-Sciences, Hudson, NY, United States). The maximum quantum yield of photosystem II (Fv/Fm) and the maximum photochemical yield of PSII (Fv/F0) were calculated as previously described (Genty et al., 1989). The plants were dark-adapted for 30 min before measurement. All the measurements involved the use of ten plants and were performed from 11:00 am to noon during the beginning of the flowering period. The operation of the machine and subsequent analysis were performed according to the manufacturer’s instructions.

### Transmission Electron Microscopy Analysis

Williams 82 and *Gmpgl3-1* mutant plants grown for 21 days were selected. The leaves were cut into rectangular pieces that were approximately 2 mm^*^1 mm, and the plant materials were vacuum fixed in 2.5% glutaraldehyde solution with 0.2 mol of phosphate buffer. The samples were postfixed for 3 h in 1% osmium tetroxide at 4°C. The samples were then treated according to previously described methods (Kowalewska et al., 2016). Ultrathin sections were obtained using an MT-X (RMC, Tucson, AZ, United States) ultramicrotome and stained with uranyl acetate for 20 min followed by lead citrate for 10 min. Observations of the samples and recording of images were performed using a Hitachi H-7650 electron microscope (Tokyo, Japan).

### CRISPR/Cas9 Vector Construction and Soybean Transformation

To obtain *GmTic110a*-knockout plants, the CRISPR/Cas9 gene editing system for targeted genome modification of plants was used (Shan et al., 2013). Several 20-nt single-guide RNAs (sgRNAs) highly specific for Cas9 target sites were identified using the web-based tool CRISPR-P version 2.0^2^ (Lu et al., 2017). A pair of 24-bp long oligonucleotides (5′-GATTGCGGCGGCTGGATACGGCCT-3′ and 5′-AAACAGGCCGTATCCAGCCGCCGC-3′) specific to the *GmTic110a* sequence were annealed and cloned into a modified *VK005-04-soU6-2-GmUbi3* knockout expression vector (Du et al., 2016). The resulting recombinant plasmid (*VK005-GmTic110a*) was introduced into *Agrobacterium tumefaciens EHA105*, which was then used to transform Williams 82 cotyledonary explants (Zhao et al., 2016). Three independent *GmTic110a*-knockout transgenic plants were obtained for further phenotypic analysis.

### Analysis of the Expression Profile of the *GmTic110a* Gene

New leaves at the VE (emergence) stage; stem tips, stems, and roots at the V1 (first unrolled trifoliate leaf) stage; leaves and flowers at the R1 (beginning bloom) stage; leaves and flowers at the R2 (full bloom) stage; and leaves at the R3 (beginning of pod development) stage were collected. Total RNA was subsequently extracted using TRIzol reagent (Tiangen, Lot 118,721; China) according to the manufacturer’s instructions. The integrity of the RNA was determined through agarose gel electrophoresis, and complementary DNA (cDNA) was synthesized using 5 μg of RNA with oligo(dT)_18_ primers and Moloney murine leukemia virus (M-MLV) reverse transcriptase (TransGen Lot N31204; China) according to the manufacturer’s protocol. Relative transcript levels of *GmTic110a* were analyzed through real-time quantitative PCR (qRT–PCR) on an Mx3005P instrument (Stratagene, La Jolla, CA, United States) in conjunction with SYBR Green Master Mix (Genstar Lot 9 BC01; China). The PCR parameters were 95°C for 30 s (1 cycle), 95°C for 5 s, and 60°C for 20 s (40 cycles), which was followed by a melting curve analysis at 95°C for 60 s, 55°C for 30 s, and 95°C for 30 s. The internal control gene *GmActin11* (*Glyma.18G290800*) was used for normalization of the transcript levels of *GmTic110a* in the samples (Hu et al., 2009). The relative fold differences were calculated *via* the 2^-ΔΔCt^ method. Three independent biological replicates were used to confirm the expression profiles. The specific primer pairs used are listed in Supplementary Table S4.

### Subcellular Localization Analysis

We next sought to determine the subcellular localization of GmTic110a, GmTic110a^G114A^, Gmtic110a^T805S^, GmTic110a^CR1^, GmTic110a^CR2^, and GmTic110a^CR3^ from the knockout transgenic strains. For this analysis, the full-length cDNA sequence and the mutated and knockout sequences of *GmTic110a* were cloned into pUC19-GFP (Zheng et al., 2017), and the resulting recombinant plasmid was transiently introduced into Arabidopsis (Col-0) protoplasts using 20% polyethylene glycol (Yu et al., 2015). The fluorescence signals were visualized using a Nikon C2 confocal laser scanning microscope (Japan) under a 488 nm excitation wavelength and 495–540 nm emission wavelengths to determine the subcellular localization of GmTic110a, GmTic110a^G114A^, Gmtic110a^T805S^, GmTic110a^CR1^, GmTic110a^CR2^, and GmTic110a^CR3^. Chloroplast autofluorescence was detected at wavelengths of 488 nm (excitation) and 680–700 nm (emission). Image processing was performed with ImageJ.^3^ The specific primer pairs used are listed in Supplementary Table S4.

### Luciferase Complementation Assays

Luciferase complementation assays were performed as described previously (Chen et al., 2008), with minor modifications. The coding DNA sequences (CDSs) of *GmTic110a*, *GmTic20*, *GmTic40a*, and *GmTic40b* were cloned into either a *pCAMBIA1300-NLuc* or a *pCAMBIA1300-CLuc* vector. *pCAMBIA1300-GmTic110a-NLuc*, *pCAMBIA1300-GmTic20-CLuc*, *pCAMBIA1300-GmTic40a-CLuc*, and *pCAMBIA1300-GmTic40b-CLuc* in various combinations were transferred into *Nicotiana benthamiana* leaves by *A. tumefaciens*-mediated transformation. Agrobacterium cells with N-Luc and C-Luc vectors were resuspended in infiltration buffer (pH 5.6; 10 mm 2-(N-morpholino) ethanesulfonic acid, 10 mm MgCl_2_, and 150 mm acetosyringone) to reach an optimal optical density at 600 nm in the range of 0.9 to 1. After 3 h of incubation at room temperature, the suspensions were infiltrated into the leaves of 4-week-old *N. benthamiana* plants, which were then cultivated for 2 days at 23°C. To inject tobacco leaves with 1 mmol of luciferin (115144–35-9, GoldBio) for measurements of luciferase activity, the leaves were maintained in the dark for 5 min. Images were captured using a chemiluminescence image analysis system (4600SF, Tanon). The sequences of the primers used are listed in Supplementary Table S4.

### Co-IP Assays

Co-IP assays were performed as described previously (Zhou et al., 2015; Liu et al., 2020; Wang et al., 2020), with minor modifications. The CDSs of *GmTic110a*, *GmTic20*, *GmTic40a*, and *GmTic40b* were cloned into either a *pCAMBIA1300-FLAG* or *pCAMBIA1300-HA* vector, resulting in *pCAMBIA1300-GmTic110a-FLAG*, *pCAMBIA1300-GmTic20-HA*, *pCAMBIA1300-GmTic40a-HA*, and *pCAMBIA1300-GmTic40b-HA* vectors. To measure protein–protein interactions, *A. tumefaciens* strain *EHA105* containing pairs of these constructs together with *pCAMBIA1300-GmTic110a-FLAG*, *pCAMBIA1300-GmTic20-HA*, *pCAMBIA1300-GmTic40a-HA*, and *pCAMBIA1300-GmTic40b-HA* were coinfiltrated into the leaves of 4-week-old *N. benthamiana* plants. Samples (1 g each) were then collected at 3 days after infiltration, ground in liquid nitrogen and homogenized in 1.5 ml of extraction buffer (50 mm Tris–HCl [pH 7.5], 150 mm NaCl, 1 mm EDTA [pH 8.0], 0.2% [v/v] Triton X-100, 20% [v/v] glycerol, and 1× protease inhibitor cocktail [pH 7.5]). The lysates were incubated at 4°C for 30 min and subsequently centrifuged at 15,000 × g for 30 min at 4°C. After instantaneous centrifugation, the supernatants were added to 500-μl suspensions of anti-DDDDK-tag-FLAG magnetic beads (No. M185-11R, Medical and Biological Laboratories), incubated at 4°C for 4 h, and then washed 4 times with extraction buffer. The proteins were eluted from the beads with 30 μl of 1 × Protein Loading Buffer, boiled for 5 min, and then centrifuged at 8,000 × g for 1 min at room temperature. The supernatants were electrophoretically separated *via* 10% SDS–PAGE and transferred to a nitrocellulose membrane (No. q0600003, GE Healthcare Life Sciences). Immunoblots were performed using an anti-FLAG antibody (1:5000; No. M180-5, Medical and Biological Laboratories) for probing *pCAMBIA1300-GmTic110a-FLAG* and an anti-HA antibody (1/5000, No. M180-3, Medical and Biological Laboratories) for probing *pCAMBIA1300-GmTic20-HA*, *pCAMBIA1300-GmTic40a-HA*, or *pCAMBIA1300-GmTic40b-HA*. The sequences of the primers used are listed in Supplementary Table S4.

## Results

### Phenotypic Characterization of the Chloroplast Development-Related Mutant *Gmpgl3*

**Figure 1:**
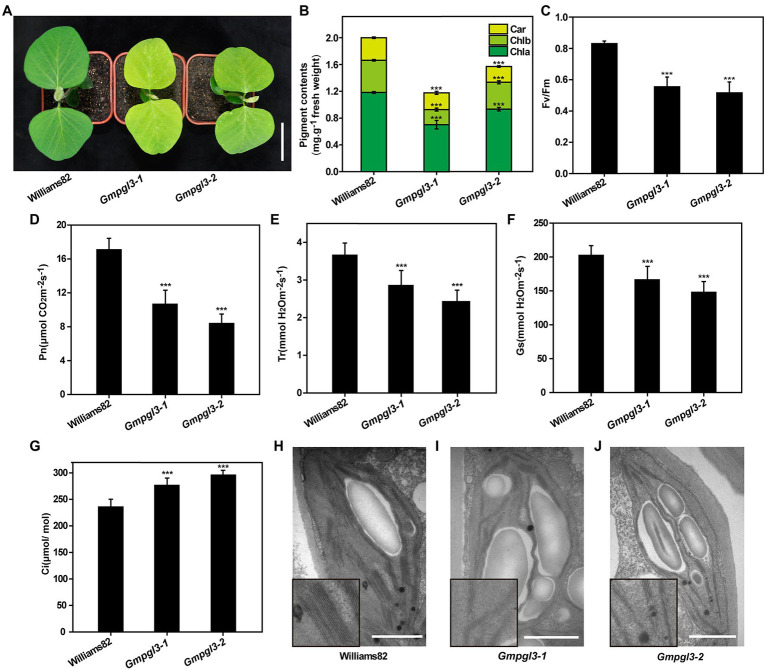
**(A)** Phenotypes of the Williams 82, *Gmpgl3-1,* and *Gmpgl3-2* mutants. Scale bars, 5 cm. **(B–G)** Pigment contents and photosynthesis parameters of the Williams 82, *Gmpgl3-1,* and *Gmpgl3-2* mutants. **(B)** Chlorophyll a (Chl a), chlorophyll b (Chl b) and carotenoid (Car). **(C)** Chlorophyll fluorescence parameter (Fv/FM). **(D)** Photosynthetic rate (Pn). **(E)** Transpiration rate (Tr). **(F)** Stomatal conductance (Gs). **(G)** Intercellular CO_2_ concentration (Ci). **(H)** Chloroplast structure in Williams 82. **(I,J)** Chloroplast structure in the *Gmpgl3-1* and *Gmpgl3-2* mutants. Scale bars, 1 μm. ^***^ represents significant differences compared with the control (Williams 82) at *p* < 0.001, and the error bars represent standard deviations.

Compared with the wild-type Williams 82, the *Gmpgl3-1* mutant showed a pale green leaf phenotype from the seedling stage to the mature stage (Figure 1A). Moreover, on the basis of their phenotypes and genotypes, we identified two allelic mutants named *Gmpgl3-2* and *Gmpgl3-3* from within the mutant library. The pale green leaf phenotype was observed for the *Gmpgl3-2* mutant (Figure 1A), while the pale green leaf phenotype was not observed for the *Gmpgl3-3* mutant (Supplementary Figure S1). We analyzed mature Williams 82, *Gmpgl3-1,* and *Gmpgl3-2* plants separately. The results showed that the overall heights of the *Gmpgl3-1* and *Gmpgl3-2* mutants were reduced by 7 and 2.5%, respectively, compared with Williams 82, while the number of nodes was reduced by 17.3%. Moreover, compared with Williams 82, the number of pods per plant for the *Gmpgl3-1* and *Gmpgl3-2* mutants was reduced by 30.8 and 39.5%, respectively; the number of grains per plant for the *Gmpgl3-1* and *Gmpgl3-2* mutants was reduced by 39.5 and 39.2%, respectively; the grain weight per plant for the *Gmpgl3-1* and *Gmpgl3-2* mutants was reduced by 50.8 and 48.2%, respectively; and the 100-seed weight for the *Gmpgl3-1* and *Gmpgl3-2* mutants was reduced by 18.1 and 14.8%, respectively (Supplementary Table S1).

Because the *Gmpgl3-1* and *Gmpgl3-2* mutants exhibited a pale green leaf phenotype throughout the growth period, the chlorophyll and carotenoid contents of Williams 82, *Gmpgl3-1*, and *Gmpgl3-2* leaves were measured spectrophotometrically, and the results showed that both chlorophyll a and chlorophyll b contents in the *Gmpgl3-1* and *Gmpgl3-2* mutants were significantly lower than those in the wild type (Figure 1B). This finding indicates that the *GmTic110a* gene mutation may affect the stability of Chl a/b. The chlorophyll content of the *Gmtic110a* mutant was reduced by 44.2%. The chloroplast fluorescence Fv/Fm values of *Gmpgl3-1* and *Gmpgl3-2* were significantly lower than those of Williams 82, which showed that the photosynthetic efficiency of *Gmpgl3-1* and *Gmpgl3-2* was lower than that of Williams 82 (Figure 1C). The photosynthetic rate (Pn) of the *Gmpgl3-1* and *Gmpgl3-2* mutants was reduced by 37.7 and 50.9%, respectively, compared with that of Williams 82. In addition, compared with those of the wild type, the stomatal conductance (Gs) of the *Gmpgl3-1* and *Gmpgl3-2* mutants decreased by 22 and 33.7%, respectively, the transpiration rate (Tr) of the *Gmpgl3-1* and *Gmpgl3-2* mutants decreased by 17.9 and 26.9%, respectively, and the intercellular CO_2_ concentration of the *Gmpgl3-1* and *Gmpgl3-2* mutants increased by 17.3 and 25.6%, respectively (Figures 1D–G). Taken together, these results showed that the decrease in the chlorophyll content in the *Gmpgl3-1* and *Gmpgl3-2* mutants significantly affected the photosynthetic ability of the leaves of those plants.

Compared with that in the wild-type (Williams 82) chloroplasts, the number of starch grains in the chloroplasts of the *Gmpgl3-1* and *Gmpgl3-2* mutants increased, and the basal thylakoids became thinner (Figures 1H–J). Because there are photosynthetic pigment components on the thylakoid membrane and because photosynthesis mainly occurs within thylakoids, both the chlorophyll a and chlorophyll b contents in the mutants were significantly reduced, which led to thinning of the basal thylakoid membrane and a reduction in the photosynthesis rate.

### Genetic Mapping of the *Gmpgl3-1* Mutation Locus From the F_2_ Population

**Figure 2:**
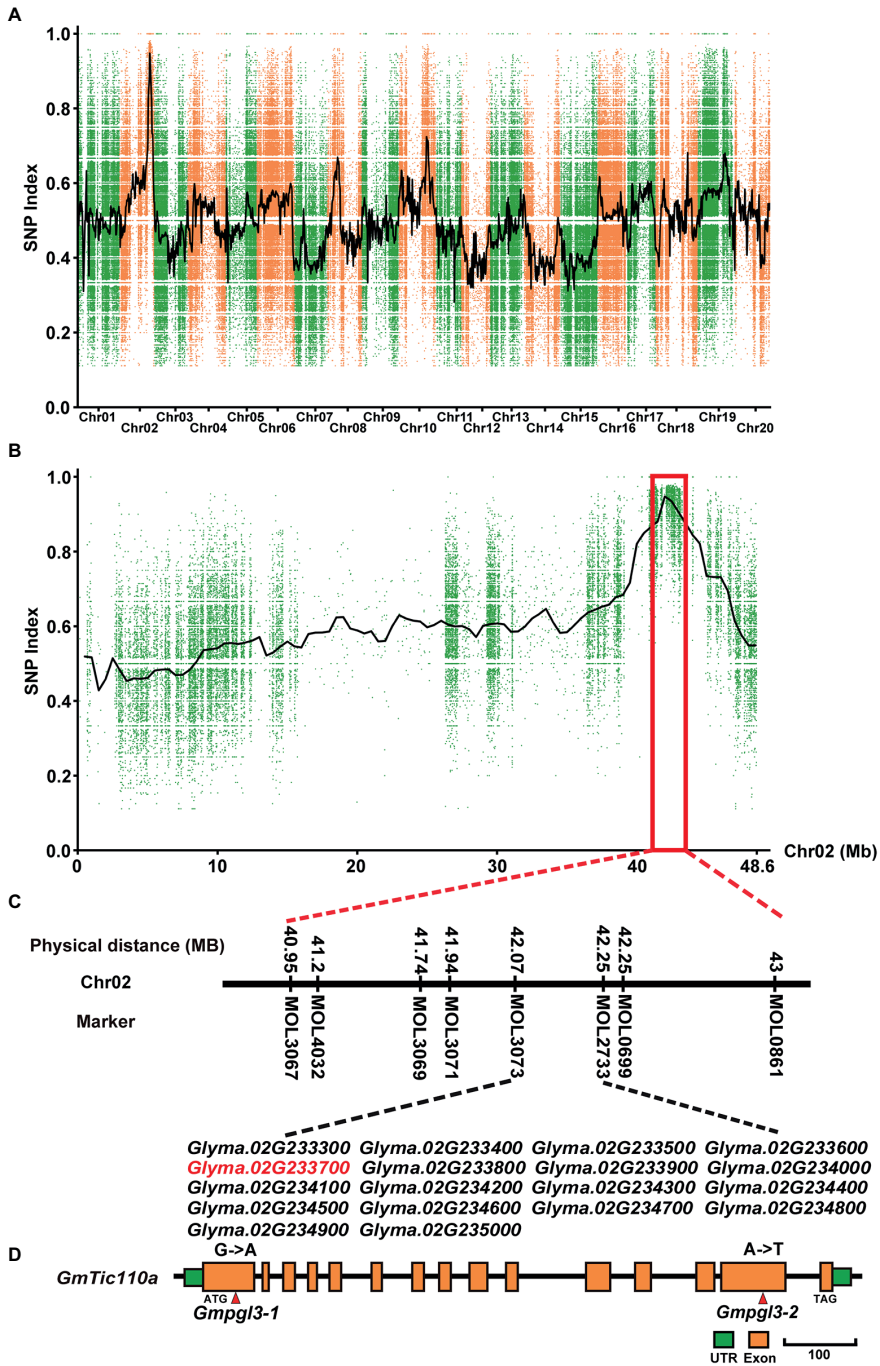
Map-based cloning of the GmTic110a locus. **(A)** SNP index plot of all chromosomes of the F_2_ plants. **(B)** SNP index plots of chromosome 02 of the *Gmpgl3a* mutant from the F_2_ population. **(C)** Physical position of the *GmTic110a* candidate gene. **(D)** Schematic diagram showing the structure of *GmTic110a*. The red lines indicate mutation sites within the *GmTic110a* gene in the two mutant lines.

The *Gmpgl3-1* mutant was crossed with Hedou 12 to generate a segregating population for mapping the *Gmtic110a* gene. The F_2_ segregating population comprised 537 plants: 423 wild-type plants and 114 mutant plants. A 3:1 segregation ratio was observed for the three F_2_ segregating populations (*χ*^2^ = 1.89, *df* = 1, *p* = 0.17; Supplementary Table S2), indicating that the *Gmpgl3-1* mutant is the result of a single recessive gene. The F_2_ population was used to identify the *Gmpgl3-1* locus. A total of 60 INDEL markers covering all 20 chromosomes were used for mapping, and the mapping results showed that *Gmpgl3-1* was restricted to a 2-Mb region (41–43 Mb) on chromosome 02 (Figures 2A,B). To finely map the *Gmpgl3-1* locus, we developed 7 INDEL markers, that is, MOL3067, MOL4032, MOL3069, MOL3071, MOL3073, MOL2733, and MOL0699; the *Gmpgl3-1* locus was further narrowed down to a 0.44-Mb region between 41.79 Mb and 42.23 Mb on chromosome 02, which harbors 18 annotated genes (Figure 2C). To identify the causal mutation, the DNA from 40 F_2_ individuals carrying the *Gmpgl3-1* mutation under homozygous conditions and the DNA of 50 F_2_ individuals exhibiting the wild-type phenotype were pooled into a *Gmpgl3-1* bulk and a Williams 82 bulk for further BSA. The *Gmpgl3-1* mutant was resequenced to a depth of approximately 30× using an Illumina HiSeq 2000 device. We identified a G_−341_ to A_−341_ transition in the first exon of *Glyma.02G233700* (Figure 2D), which caused a non-synonymous substitution of Gly_−114_ to Asp_−114_ in the predicted protein. No other mutations were discovered among the 18 genes in the candidate *Gmpgl3-1* genomic region (Figure 2C; Supplementary Table S3). However, a single-base mutation (A–2413 to T–2413) was identified in the 14th exon of the *GmTic110a* gene of the *Gmpgl3-2* mutant (Figure 2D). The expression level of *Glyma.02G233700* decreased in the *Gmpgl3-1* and *Gmpgl3-2* mutants (Supplementary Figure S2), suggesting that *Glyma.02G233700* is the *GmTic110a* gene.

### GmTic110a Encodes an Inner Chloroplast Membrane Protein

**Figure 3:**
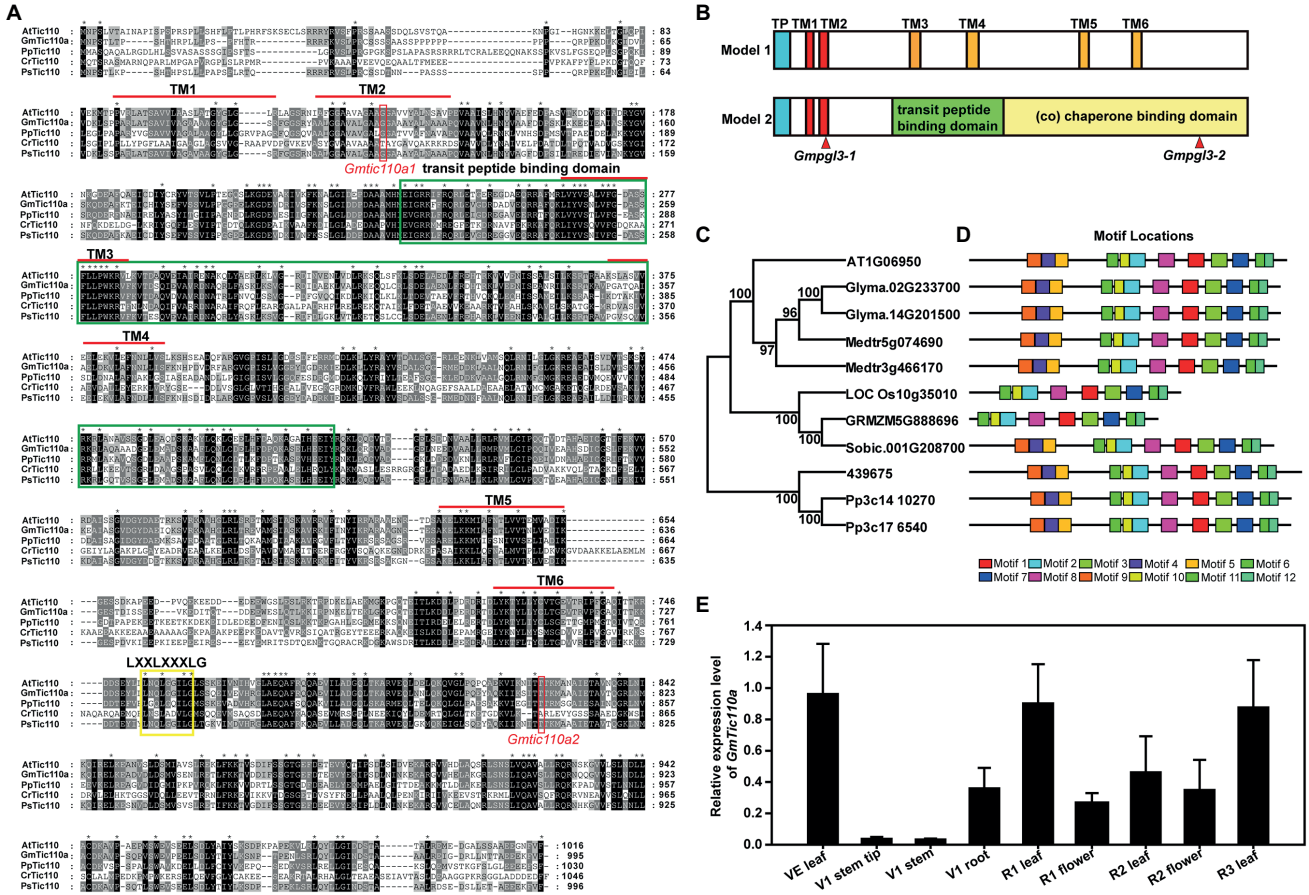
**(A)** Multiple sequence alignments of the Tic110 protein in *Arabidopsis thaliana* (At), *Glycine max* (Gm), *Physcomitrella patens* (Pp), *Chlamydomonas reinhardtii* (Cr), and *Pisum sativum* (Ps). The dark region represents identical amino acids, and the grey region represents similar amino acids. **(B)** Schematic diagram representations of the two structural models of the Tic110 protein. In terms of the locations of the proposed TM domains, the red boxes represent TM1 and TM2, the orange boxes represent TM3 to TM6, the blue boxes represent transit peptides (TPs), the green box represents TP binding, and the yellow box represents a co-domain. **(C)** Phylogenetic trees based on the multiple sequence alignments of the Tic110 proteins. Bootstrap values from 1,000 replicates are indicated at each node. **(D)** Conserved motifs of Tic110 proteins in *G. max*, *A. thaliana*, *Medicago sativa*, *Oryza sativa*, *Zea mays*, *Sorghum bicolor*, *Selaginella tamariscina*, and *P. patens* were identified using the MEME search tool. Different motifs (1–12) are represented by boxes with different colors. **(E)** Tissue-specific expression profiles were determined *via* qRT–PCR.

BLAST searches revealed that GmTic110a, which encodes a chloroplast inner membrane protein, is highly homologous to the soybean GmTic110b protein (Glyma.14G201500; 98.4% amino acid similarity) and the Arabidopsis Tic110 protein (At1G06950; 84.3% amino acid similarity). In Medicago, the gene with the highest homology to *GmTic110a* is Medtr5g074690, whose sequence is 90.5% identical to that of *GmTic110a* and 86.4% identical to that of Medtr3g466170. Both of their proteins are 994 and 985 amino acids in length. Amino acid sequence analysis resulted in the generation of a phylogenetic tree composed of the *GmTic110a* homologous gene and its homologs from dicotyledonous plant species (*G. max*, *A. thaliana*, and *Medicago sativa*), monocotyledonous plant species (*Oryza sativa*, *Zea mays*, and *Sorghum bicolor*), *Selaginella tamariscina*, and *Physcomitrella patens* (Figure 3C). The results suggest that two GmTic110 homologs are evolutionarily conserved among plant species and share a common genomic structure in the observed plant species. The results of MEME analysis showed that *GmTic110a* contains 12 conserved motifs (Figure 3D). The mutation site of the *Gmpgl3-1* mutant is located within the conserved TM2 domain, the mutation site of the *Gmpgl3-2* mutant is located within the conserved chaperone-binding (co) domain, and the *Gmpgl3-3* mutant site is located within the terminal TM1 domain (Figures 3A,B).

Using qRT–PCR, we examined the *GmTic110a* expression patterns in new leaves at the VE stage; tips, stems, and roots at the V1 stage; leaves and flowers at the R1 stage; leaves and flowers at the R2 stage; and leaves at the R3 stage. *GmTic110a* was slightly expressed in the tips and stems and had low expression in the roots and flowers. The highest expression levels were detected in the leaves at various stages (new leaves at the VE stage, leaves at the R1 stage, leaves at the R2 stage, and leaves at the R3 stage; Figure 3E), indicating that *GmTic110a* may play an important role in leaf development and regulatory processes. These results also explained why the *GmTic110a* mutation severely affected leaf growth at various stages.

### CRISPR/Cas9-Mediated *GmTic110a* Gene Editing of Transgenic Plants

**Figure 4:**
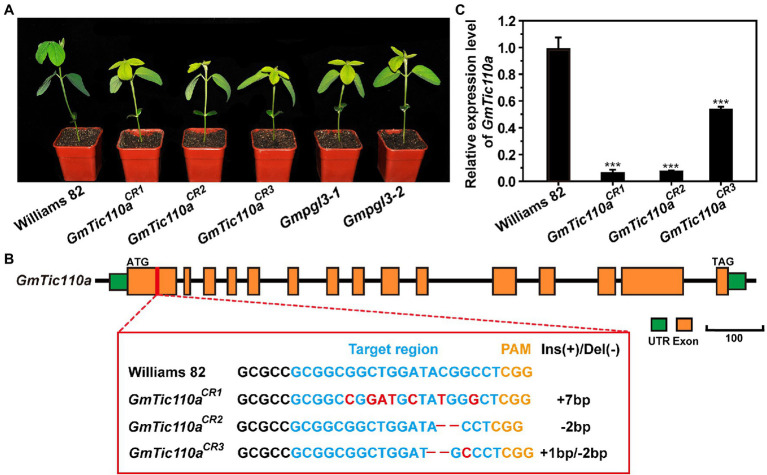
**(A)** Phenotypes of Williams 82, the *Gmpgl3* mutant, and CRISPR/Cas9-edited plants (*GmTic110a^CR1^*, *GmTic110a^CR^*,*^2^* and *GmTic110a^CR3^*). Scale bar = 1 cm. **(B)** sgRNA target sequences of Williams 82, *GmTic110a^CR1^*, *GmTic110a^CR2^*, and *GmTic110a^CR3^*. The sgRNA target sequence is shown in blue letters, and the protospacer-adjacent motif (PAM) site is shown in yellow letters. The red letters indicate a single-base substitution. **–** indicates a deletion of the corresponding nucleotide. **(C)** Relative expression of the *GmTic110a* gene in unifoliate leaves of Williams 82, the *Gmpgl3* mutant, and CRISPR/Cas9-edited plants. The asterisks indicate statistically significant differences, as determined by Student’s *t*-test (^***^, p < 0.001), and the error bars represent the standard deviations.

To confirm whether *Gmpgl3-1* was the *GmTic110a* gene, loss-of-function transgenic lines were generated by inducing mutations in the *GmTic110a* gene using the CRISPR/Cas9 system. The resulting CRISPR/Cas9-induced mutations in three separate mutants led to the development of a *GmTic110*-specific mutant phenotype (Figure 4A). The results showed that the phenotypes of *GmTic110a^CR1^*, *GmTic110a^CR2^*, and *GmTic110a^CR3^* were the same as those of *Gmpgl3-1* and *Gmpgl3-2*. The *GmTic110a^CR1^* mutant contains a 7-bp substitution corresponding to the CDS of the *GmTic110a* gene from the 261st bp to the 273rd bp. The *GmTic110a^CR2^* mutant had a 2-bp deletion from the 271st to the 272nd bp, and the *GmTic110a^CR3^* mutant had a 2-bp deletion at 269th bp of the CDS of *GmTic110a* and a 1-bp substitution (Gly to Ala) at the 272nd bp (Figure 4B). These data suggested that the *GmTic110a^CR1^*, *GmTic110a^CR2^* and *GmTic110a^CR3^* mutants have strong alleles and that complete loss of *Gmpgl3* function strongly influences soybean development (Supplementary Table S1). Moreover, the expression levels of *Glyma.02G233700* decreased in *GmTic110a^CR1^*, *GmTic110a^CR2^*, and *GmTic110a^CR3^* (Figure 4C). These results further suggested that *Glyma.02G233700* is the *GmTic110a* gene.

### The GmTic110a Protein Localizes to the Chloroplast Inner Membrane

**Figure 5:**
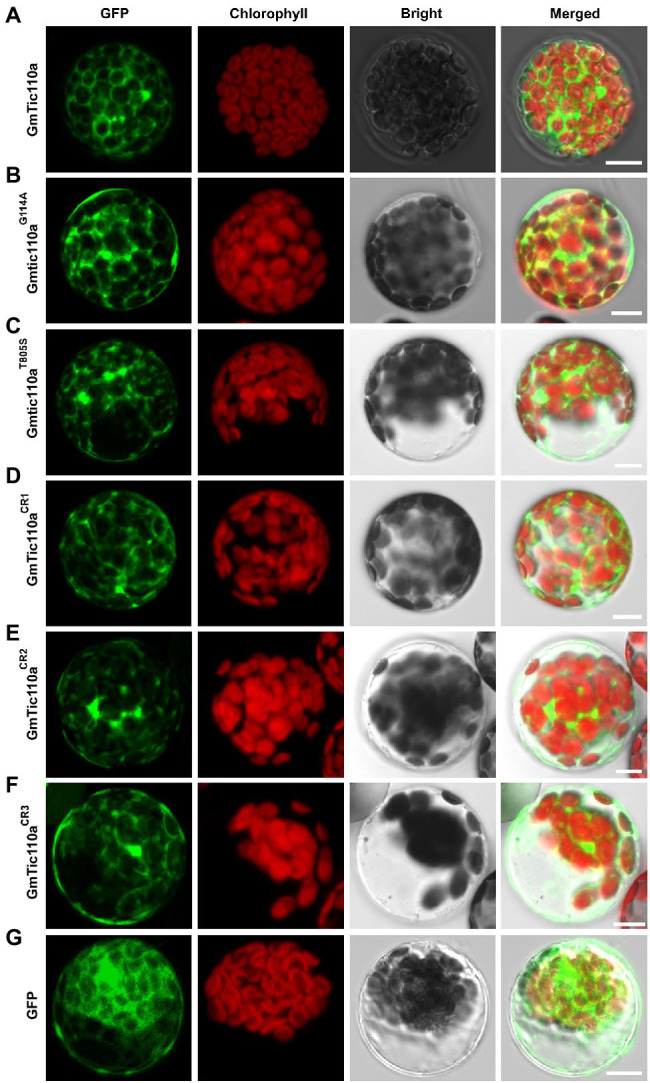
Subcellular localization of GmTic110a, GmTic110a^G114A^, Gmtic110a^T805S^, GmTic110a^CR1^, GmTic110a^CR2^, and GmTic110a^CR3^. **(A-G)** Transient expression of GFP-GmTic110a, GFP-GmTic110a^G114A^, GFP-Gmtic110a^T805S^, GFP-GmTic110a^CR1^, GFP-GmTic110a^CR2^, GFP-GmTic110a^CR3^ and GFP in Arabidopsis protoplasts. GFP, GFP fluorescence; Chlorophyll, chlorophyll autofluorescence; Bright, bright field. Merged, merged image of GFP fluorescence, chlorophyll autofluorescence and bright field images. Scale bars = 10 μm.

To confirm the subcellular localization of GmTic110a, the colocalization of green fluorescent proteins (*GmTic110a*-GFP) and the AtPIC1-mCherry marker protein (localization to the inner envelope of chloroplasts; Duy et al., 2007) was analyzed. As shown in Supplementary Figure S3, the *GmTic110a* protein targeted the inner chloroplast membrane (Figure 5A). At the same time, a single transformation of an empty vector that contained GFP was used as a positive control, and the results revealed that the fluorescent signal of the empty vector was detected throughout the entire cell protoplast (Figure 5G). The results were consistent with the bioinformatics predictions of the subcellular localization of *GmTic110a* (Supplementary Table S5). To determine the effects of *Gmpgl3* mutations on protein localization, we transiently transformed PUC19-GFP-GmTic110a^G114A^, PUC19-GFP-Gmtic110a^T805S^, PUC19-GFP-GmTic110a^CR1^, PUC19-GFP-GmTic110a^CR2^, and PUC19-GFP-GmTic110a^CR3^ into Arabidopsis protoplast cells. The results showed that proteins resulting from a mutated or knocked out *GmTic110a* gene were located in the inner chloroplast membrane, but the GFP fluorescence signal remained diffuse (Figures 5B–F), indicating that *GmTic110a* mutation or knockout altered the structure of the *GmTic110a* protein, thereby affecting the subcellular localization of the protein.

### GmTic110a Interacts With GmTic20, GmTic40a, and GmTic40b

**Figure 6:**
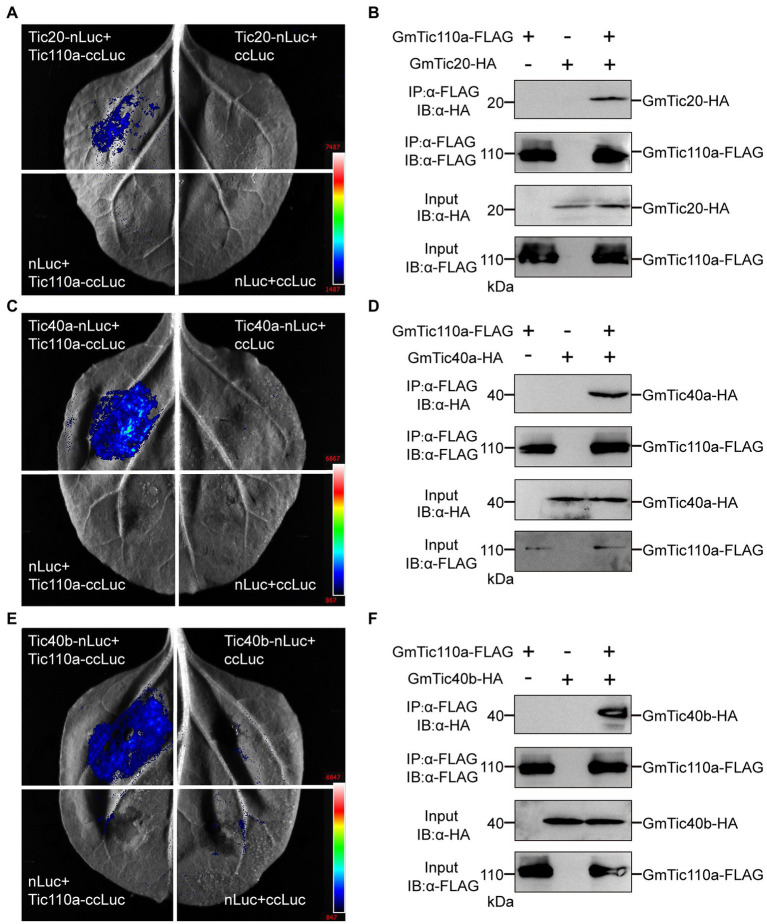
*GmTic110a* interacts with GmTic20, GmTic40a, and GmTic40b. **(A,C,E)** Luciferase complementation assay showing that *GmTic110a* interacts with GmTic20, GmTic40a, and GmTic40b in *Nicotiana benthamiana*. Luciferase activity was detected 3 days after injection. **(B,D,F)** Interactions with GmTic20, GmTic40a, and GmTic40b in *N. benthamiana* according to a Co-IP assay. Immunoblots of the total protein extracts (20% input) and the immunoprecipitation product were performed using an anti-HA antibody (a-HA) or an anti-FLAG antibody (a-FLAG), respectively.

Previous studies have shown that AtTic110 mediates transport across the inner membrane *via* interactions with AtTic40 and other proteins (Kovacheva et al., 2005; Chiu and Li, 2008; Yuan et al., 2021). Because the GmTic40 protein contains a highly conserved transmembrane (TM) motif, we investigated whether GmTic40 functions in conjunction with AtTic40. We used split luciferase complementation assays to confirm whether *GmTic110a* proteins could interact with GmTic20, GmTic40a, and GmTic40b (Figures 6A,C,E). The interactions of *GmTic110a* with GmTic20, GmTic40a, and GmTic40b were verified by co-IP analyses. As shown in Figure 6, we transiently coexpressed *GmTic110a* with GmTic20, GmTic40a, and GmTic40b in *N. benthamiana* leaves. Total proteins were isolated, after which they and anti-FLAG magnetic beads were incubated together to immunoprecipitate anti-FLAG. The results showed that GmTic110a, GmTic20, GmTic40a, and GmTic40b were present in the immunoprecipitate (Figures 6B–F), indicating that *GmTic110a* could interact with GmTic20, GmTic40a, and GmTic40b *in vivo*.

## Discussion

Currently, there are two hypotheses concerning the Arabidopsis Tic110 protein structure. Hypothesis 1 proposes that the Tic110 protein is composed of 6 TM domains (TM1, TM2, TM3, TM4, TM5, and TM6), while hypothesis 2 proposes that the Tic110 protein consists of 2 TM domains, one transit peptide-binding domain and 1 co-domain. We compared the amino acid sequences of the Tic110 proteins of *A. thaliana*, *G. max*, *P. patens*, *C. reinhardtii*, and *P. sativum* and identified six TM domains, transit peptide-binding domain domains, and co-domains (Figure 3A). By analyzing the structural site of the *Gmpgl3* mutant, we confirmed that the mutation site of the *Gmpgl3-1* mutant lies in the TM domain of TM2 (position 113 aa), the mutation site of the *Gmpgl3-2* mutant lies in the co-domain (amino acid position 805 aa), and the mutation site of the *Gmpgl3-3* mutant lies in the TM domain of TM1 (amino acid position 94 aa; Figure 3A). Phenotypic observations revealed that both the *Gmpgl3-1* and *Gmpgl3-2* mutants showed a pale green leaf phenotype (Figure 1A); *Gmpgl3-3* did not display a pale green leaf phenotype (Supplementary Figure S1). We speculated that the site variation of the *Gmpgl3-1* mutant affected the TM domain of TM2, indicating that it is the key site of protein function. In contrast, the mutation site of the *Gmpgl3-3* mutant is an amino acid at the end of the TM domain of TM1, and the mutation did not cause phenotypic abnormalities, indicating that this site is not a key site of the TM domain. The phenotype of the *Gmpgl3-2* mutant is similar to that of *Gmpgl3-1*, as both show a pale green leaf phenotype. The gene mutation site of *Gmpgl3-2* is in the co-domain. Mutations in this site may affect the *GmTic110a* gene and its function, which may impact the development of chloroplasts. The *GmTic110a* protein was knocked out by CRISPR/Cas9 technology, and the resulting three plants with point mutations or deletions in the TM1 domain all showed similar phenotypes, which further confirmed that the phenotype was caused by a mutation in this gene. The knockout experiment using CRISPR/Cas9 technology also showed that the protein structural change due to the mutation is the main cause of the pale green leaf phenotype.

A protein must be in a suitable subcellular location to perform its function. Therefore, studying protein subcellular location is highly important for understanding protein function. This study used the online tool WoLF PSORT (Horton et al., 2006) and Target-P 1.1 Server (Emanuelsson et al., 1999) to predict the subcellular location of *GmTic110a*, which showed that the N-terminal domain contains chloroplast transit peptides, indicating that TIC110a is located on chloroplasts (Supplementary Table S5). To confirm whether the GmTic110a protein is located on the chloroplast, we extracted protoplasts from *A. thaliana* and transiently expressed GmTic110a-GFP constructs. The results showed that GmTic110a is located mainly on the chloroplast membrane (Figure 5A), which is consistent with the results predicted by the online tool. These results also indicated that the soybean GmTic110a protein is on the inner membrane of the chloroplast and is responsible for the TM transport of proteins into the chloroplast to perform normal functions. It was reported that the Arabidopsis *tic110* mutant exhibits pale green leaves and an albino phenotype. Trypsin digestion was used to prove that the Arabidopsis TIC110 fusion protein is located on the chloroplast membrane (Duy et al., 2007; Yuan et al., 2021). In this study, we confirmed that Tic110 proteins of soybean and Arabidopsis have the same subcellular localization. To confirm that the function of the GmTic110a protein is related to its specific membrane localization, we compared the GmTic110a protein localization patterns between mutant, deletion, and wild-type *GmTic110a* proteins. Point mutations or deletions of the GmTic110a protein alter the normal localization of the protein, and the protein is diffusely distributed throughout the chloroplast membrane (Figures 5B–F). Mutation or deletion of the TM1 domain of GmTic110a affected protein localization and normal function in the cells. The mutation or deletion position occurs after the leader peptide (amino acids 33–56 aa) in TM1, in the TM2 domain or in the co-domain, which does not affect the positioning of the *GmTic110a* protein in the chloroplast membrane. Point mutations or deletions in these conserved domains may affect the TM positioning or interactions with the protein, thereby affecting the chloroplast membrane localization and function of the GmTic110a protein.

Tic20, Tic110, and Tic40 are considered components of the TIC import machinery in the chloroplast; however, Kikuchi et al. (2013) reported that only Tic56, Tic100, and Tic214 were isolated from the 1-megadalton complex when using a tagged form of Tic20. Ramundo et al. (2020) also demonstrated that the TIC complex contains Tic20, Tic56, Tic100, and Tic214 by combining transcriptomic, biochemical, and genetic tools in the green alga Chlamydomonas, indicating that the complex is widely conserved among photosynthetic organisms (Ramundo et al., 2020). This result conflicts with our finding of the *GmTic110a* interaction with GmTic20 in the above studies, even though a similar interaction was also reported previously (Kouranov et al., 1998; Chen et al., 2002; Inaba et al., 2003). We suspected that these results were more likely caused by method limitations, and further physical interaction experiments will clear this confusion in the future. It is still unclear how Tic110 and Tic40 interact with the 1-megadalton complex, as they might be recruited to coordinate chaperone functions during later stages and/or are only required for the import of some preproteins (Lee and Hwang, 2018; Nakai, 2018; Thomson et al., 2020). Lee et al. reported that there were no differences in the import of preprotein *via* the wild-type transit peptide between *tic40* and wild-type protoplasts of *Arabidopsis thaliana*, while the import of N-terminal mutants of the RbcS protein (RbcS-nt) was dependent on Tic40; however, HA (hemagglutinin)-tagged Tic40 showed an intermediate form present in the stroma of *tic40* protoplasts (Lee and Hwang, 2019). In this study, we determined that GmTic110a interacted with GmTic20, GmTic40a, and GmTic40b in tobacco leaves (Figure 6); however, GmTic110a may also interact with other unknown partners. Thus, in the future, additional biochemical experiments will be performed to evaluate whether there is a direct interaction between them.

## Data Availability Statement

The datasets presented in this study can be found in online repositories. The names of the repository/repositories and accession number(s) can be found in the article/supplementary material.

## Author Contributions

XF, SY, and HY designed the research. HY, QW, ZZ, TW, and XY performed the experiments. XZ, YY, and JL analyzed the data. HY, QW, and XF wrote the manuscript. All authors contributed to the article and approved the submitted version.

## Funding

This work was supported by the National Natural Science Foundation of China (grant nos. 31700213 and U21A20215), Science and Technology Development Plan Project of Jilin Province of China (grant nos. 20210302005NC), and Zhejiang Lab (grant nos. 2021PE0AC04).

## Conflict of Interest

The authors declare that the research was conducted in the absence of any commercial or financial relationships that could be construed as a potential conflict of interest.

## Publisher’s Note

All claims expressed in this article are solely those of the authors and do not necessarily represent those of their affiliated organizations, or those of the publisher, the editors and the reviewers. Any product that may be evaluated in this article, or claim that may be made by its manufacturer, is not guaranteed or endorsed by the publisher.

## Footnotes

^1^ http://www.ncbi.nlm.nih.gov/

^2^ http://crispr.hzau.edu.cn/cgi-bin/CRISPR2/CRISPR

^3^ http://rsb.info.nih.gov/ij/

## Abbreviations

TOC: Translocon at the Outer Envelope Membrane of Chloroplasts
TIC: Translocon at the Inner Envelope Membrane of Chloroplasts
EMS: Ethyl Methanesulfonate
BSA: Bulked Segregation Analysis
SNP: Single-Nucleotide Polymorphism
NCBI: National Center for Biotechnology Information
MEGA 7.0: Molecular Evolutionary Genetics Analysis Software, Version 7.0
MEME: Multiple Expectation Maximization for Motif Elicitation
Pn: Photosynthetic Rate
Gs: Stomatal Conductance
Ci: Intercellular CO_2_ Concentration
Tr: Transpiration Rate
F0: Initial Fluorescence
Fm: Maximal Fluorescence
Fv: Variable Fluorescence
cDNA: Complementary DNA
qRT–PCR: Real-Time Quantitative PCR
