# Supplementary material for: Genetic Mapping of the Gmpgl3 Mutant Reveals the Function of GmTic110a in Soybean Chloroplast Development
Source: Front Plant Sci. 2022 May 26;13:892077. doi: 10.3389/fpls.2022.892077 (PMC9178232; doi:10.3389/fpls.2022.892077)
Supplement: Supplementary file 1 [file Data_Sheet_1.docx]

Supplementary Material

# Supplementary Figures


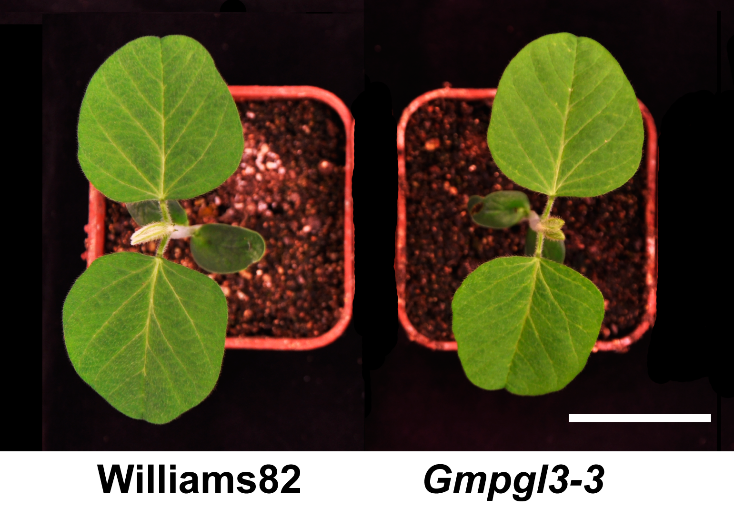


**Supplementary Figure S1.** Phenotypes of the Williams 82 and *Gmpgl3-3* mutant.


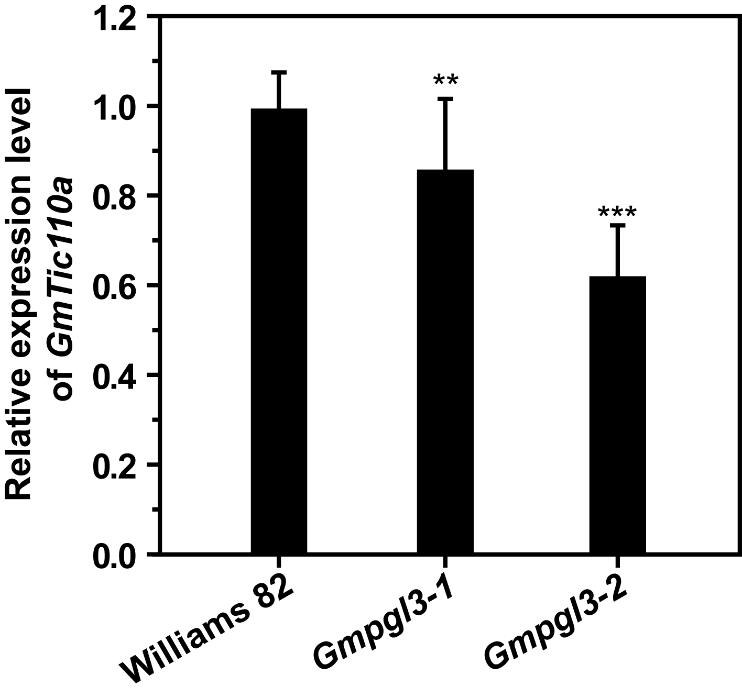


**Supplementary Figure S2.** Relative expression of the *GmTic110a* gene in unifoliate leaves of the Williams 82, *Gmpgl3-1* and *Gmpgl3-2* mutants. The asterisks indicate statistically significant differences, as determined by Student's t test (***, P<0.001; **, P<0.01), and the error bars represent the standard deviations.


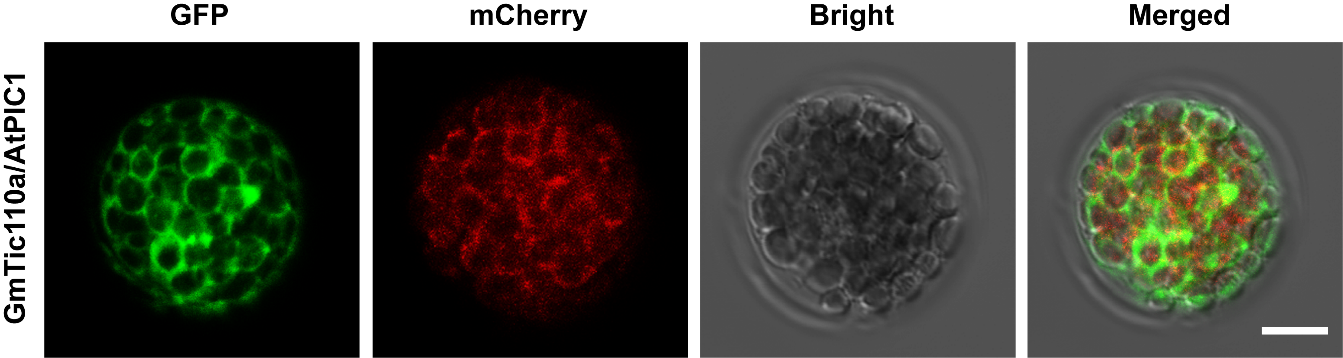


**Supplementary Figure S3.** Subcellular colocalization of GmTic110a protein and AtPIC1 protein. Transient expression of GFP-GmTic110a and GFP-AtPIC1 in Arabidopsis protoplasts. GFP, GFP ﬂuorescence; mCherry, Red ﬂuorescence; Bright, bright ﬁeld. Merged, merged image of the GFP fluorescence, red ﬂuorescence and bright field images. Scale bars = 10 µm.

# Supplementary Tables

**Supplementary Table S1.** Comparison of plant height, main stem node number, internode length and other yield traits.

|  | **Williams82** | ***Gmpgl3-1*** | ***Gmpgl3-2*** | ***GmTic110^CR^-1*** | ***GmTic110^CR^-2*** | ***GmTic110^CR^-3*** |
| --- | --- | --- | --- | --- | --- | --- |
| Plant height (cm) | 112.1±5.1 | 104.3±7.2** | 109.3±6.3 | 91.9±4.9*** | 96.8±6.8*** | 97.0±4.8*** |
| Main stem node number | 26.6±1.2 | 22.0±2.8*** | 22.0±2.1*** | 23.6±1.8*** | 22.0±1.8*** | 24.8±1.5** |
| Number of pods per plant | 103.8±18.2 | 71.8±11.5*** | 62.8±10.2*** | 78.6±8.2*** | 79.1±8.9*** | 77.1±11.7*** |
| Grain number per plant | 237.4±38.1 | 143.6±23.0*** | 144.4±23.6*** | 165.1±17.3*** | 166.1±18.7*** | 158.1±23.9*** |
| Grain weight per plant (g) | 56.1±10.0 | 27.6±4.4*** | 29.0±5.1*** | 30.0±3.4*** | 31.4±3.3*** | 29.4±4.2*** |
| 100-grain weight (g) | 23.6±0.5 | 19.3±1.9*** | 20.1±1.0*** | 18.2±0.7*** | 18.9±0.6*** | 18.6±0.6*** |

Mature soybean were used for measurements. Data are the average of 10 samples (± SD). **, P<0.01; ***, P<0.001.

**Supplementary Table S2.** Chi-square test for segregation ration of F_2_ generation

(*Gmpgl3-1*×Hedou 12)

| **Crosses**  **(*Gmpgl3-1*×Hedou 12)** | **F_1_** | **Number of F_2_ Plants** | | | ***χ^2^*** | ***df*** | ***p*** |
| --- | --- | --- | --- | --- | --- | --- | --- |
|  |  | **Wild Type Mutant Total** | | |  |  |  |
| *SI8011* | Normal | 423 | 114 | 537 | 1.89 | 1 | 0.17 |

**Supplementary Table S3.** Predicted genes present within the sequenced region

| **Gene** | **Function_type** | **Predicted Protein/Function** |
| --- | --- | --- |
| *Glyma.02G233300* | No | No functional annotation |
| *Glyma.02G233400* | No | No functional annotation |
| *Glyma.02G233500* | Nonsynonymous | Pseudouridylate synthase / Uracil hydrolyase // tRNA pseudouridine (38-40) synthase |
| *Glyma.02G233600* | No | Pseudouridylate synthase / Uracil hydrolyase // tRNA pseudouridine (38-40) synthase |
| ***Glyma.02G233700*** | **Nonsynonymous** | **Chloroplast envelope transporter (Tic110)** |
| *Glyma.02G233800* | No | Peroxidase |
| *Glyma.02G233900* | No | Peroxidase |
| *Glyma.02G234000* | No | Peroxidase |
| *Glyma.02G234100* | No | PTHR13073 - GCN5-RELATED |
| *Glyma.02G234200* | No | Peroxidase |
| *Glyma.02G234300* | No | PTHR23315//PTHR23315:SF117 - BETA CATENIN-RELATED ARMADILLO REPEAT-CONTAINING |
| *Glyma.02G234400* | No | No functional annotation |
| *Glyma.02G234500* | No | tyrosyl-tRNA synthetase |
| *Glyma.02G234600* | No | SF28 - LOB DOMAIN-CONTAINING PROTEIN 13 |
| *Glyma.02G234700* | No | No functional annotation |
| *Glyma.02G234800* | No | SF167 - CBL-INTERACTING SERINE / THREONINE-PROTEIN KINASE 2 |
| *Glyma.02G234900* | No | No functional annotation |
| *Glyma.02G235000* | No | No functional annotation |

**Supplementary Table S4.** Primers used in this study

| **Primer name** | **Primer sequence (5’- 3’)** | **Annotation** |
| --- | --- | --- |
| MOL3067 F | CTTGTTGAGGCTTTGAGGATGA | Gene mapping |
| MOL3068 R | GAATCTTACGTCTGCAAGCAGAG | Gene mapping |
| MOL4032 F | CCTCTGGCAATGAACTCCTG | Gene mapping |
| MOL4033 R | CTGGGTTGGTGGACACTGAA | Gene mapping |
| MOL3069 F | CAGCAAGAATGAGATTCTTGGC | Gene mapping |
| MOL3070 R | TGACTTGAGTTTTTAATGGTGTTGC | Gene mapping |
| MOL3071 F | GCAAGTTAGTAGACAATGGTTTCAT | Gene mapping |
| MOL3072 R | TCATTATTGAAGGAAAAGAACAAGG | Gene mapping |
| MOL3073 F | GGTTTCTTTTCTGGCATGGATC | Gene mapping |
| MOL3074 R | CACATAGCAAACAGTGTTGATCCA | Gene mapping |
| MOL2733 F | AGCTCACAAGACGAATGATCAAACA | Gene mapping |
| MOL2734 R | TTGGGTTTGGAGTGGAAGAGTG | Gene mapping |
| MOL0669 F | AGTTTCAGGAGAAATCTGGCACC | Gene mapping |
| MOL0700 R | CGACTATTCAGGAATCATATGTCCA | Gene mapping |
| MOL0861 F | TGGACAAAGGTATCCCTGATGGTAG | Gene mapping |
| MOL0862 R | CAACACTCCCACAACCAGCTTAATC | Gene mapping |
| OL9168F | GATTGCGGCGGCTGGATACGGCCT | *GmTic110a*-Crispr |
| OL9169R | AAACAGGCCGTATCCAGCCGCCGC | *GmTic110a*-Crispr |
| OL8977 F | CTGTACAAGCATATGATGAACCCTTCCACACTCACCCC | Subcellular localization |
| OL8979 R | GATGAATTCGAGCTCCTAGAATACAAACTTCTCTTCCTCCGC | Subcellular localization |
| OL9129F | ATCTTGACTGAGCGTGGTTATTCC | *GmActin11* q-PCR |
| OL9130R | GCTGGTCCTGGCTGTCTCC | *GmActin11* q-PCR |
| OL9360 F | GGGTAGACTATCCAACTCTC | *GmTic110a* q-PCR |
| OL9361 R | GCACTTCCCATGAAACTGGC | *GmTic110a* q-PCR |
| OL9368 F | GGAGTTAGCCAAGCAGTTGC | *GmTic110b* q-PCR |
| OL9369 R | GACCAACACCACGGGCAAAG | *GmTic110b* q-PCR |
| OL10560F | ACGGGGGACGAGCTCGGTACCATGAACCCTTCCACACTCACC | *pCAMBIA1300-GmTic110a-nLuc* |
| OL10561R | CGCGTACGAGATCTGGTCGACGAATACAAACTTCTCTTCCTCCG | *pCAMBIA1300-GmTic110a-nLuc* |
| OL13155F | ACGGGGGACGAGCTCGGTACCATGATTCAAAATGGTTGCATT | *pCAMBIA1300-GmTic20-ccLuc /pCAMBIA1300-GmTic20-Ha* |
| OL13156R | AACATCGTATGGGTAGTCGACATCATATGGTATCTGGATATA | *pCAMBIA1300-GmTic20-ccLuc /pCAMBIA1300-GmTic20-Ha* |
| OL13163F | ACGGGGGACGAGCTCGGTACCATGGAGAAGCTTAACTTGGCA | *pCAMBIA1300-GmTic40a-ccLuc /pCAMBIA1300-GmTic40a-Ha* |
| OL13164R | AACATCGTATGGGTAGTCGACAGGTGAACCTACTCCAGGGAA | *pCAMBIA1300-GmTic40a-ccLuc /pCAMBIA1300-GmTic40a-Ha* |
| OL13165F | ACGGGGGACGAGCTCGGTACCATGGAGAAGCTTAACTTGGCC | *pCAMBIA1300-GmTic40b-ccLuc /pCAMBIA1300-GmTic40b-Ha* |
| OL13166R | AACATCGTATGGGTAGTCGACAGGTGAACCTACTCCAGGGAA | *pCAMBIA1300-GmTic40b-ccLuc /pCAMBIA1300-GmTic40b-Ha* |
| OL13363F | ACGGGGGACGAGCTCGGTACCATGAACCCTTCCACACTCA | *pCAMBIA1300-GmTic110a-Flag* |
| OL13364R | ATGGTCTTTGTAGTCGTCGACGAATACAAACTTCTCTTCC | *pCAMBIA1300-GmTic110a-Flag* |

**Supplementary** **Table S5.** The prediction from the subcellular localization program WoLF PSORT and Cell-PLoc 2.0.

| **Locus name** | **WoLF PSORT**  **(https://wolfpsort.hgc.jp/)** | **Cell-PLoc 2.0**  **(**[**Cell-PLoc 2.0 package (sjtu.edu.cn)**](http://www.csbio.sjtu.edu.cn/bioinf/Cell-PLoc-2/)**）** |
| --- | --- | --- |
| Glyma.02G233700 | Chlo (14) | Chloroplast |

The numbers in parenthesis indicate prior possible protein localization sites of the GmTic110a.

chlo: chloroplast;
